# 2-(4-Isopropyl-4-methyl-5-oxo-4,5-dihydro-1*H*-imidazol-2-yl)-5-methyl­nicotinic acid

**DOI:** 10.1107/S1600536812020478

**Published:** 2012-05-12

**Authors:** Li-Ping Liu, Xiao-Dan Wang, Shuang Zhang, Jin-Sheng Gao

**Affiliations:** a Engineering Research Center of Pesticides of Heilongjiang University, Heilongjiang University, Harbin 150050, People’s Republic of China

## Abstract

In the title herbicideh/phytocide, known as imaza­pic, C_14_H_17_N_3_O_3_, the pyridine and imidazole rings are almost coplanar [dihedral angle = 3.08 (5)°]. An intra­molecular O—H⋯N hydrogen bond occurs. In the crystal, an N—H⋯O hydrogen bond links mol­ecules into a chain parallel to [010].

## Related literature

For the synthesis, see: Szezepanski *et al.* (1988 ▶).
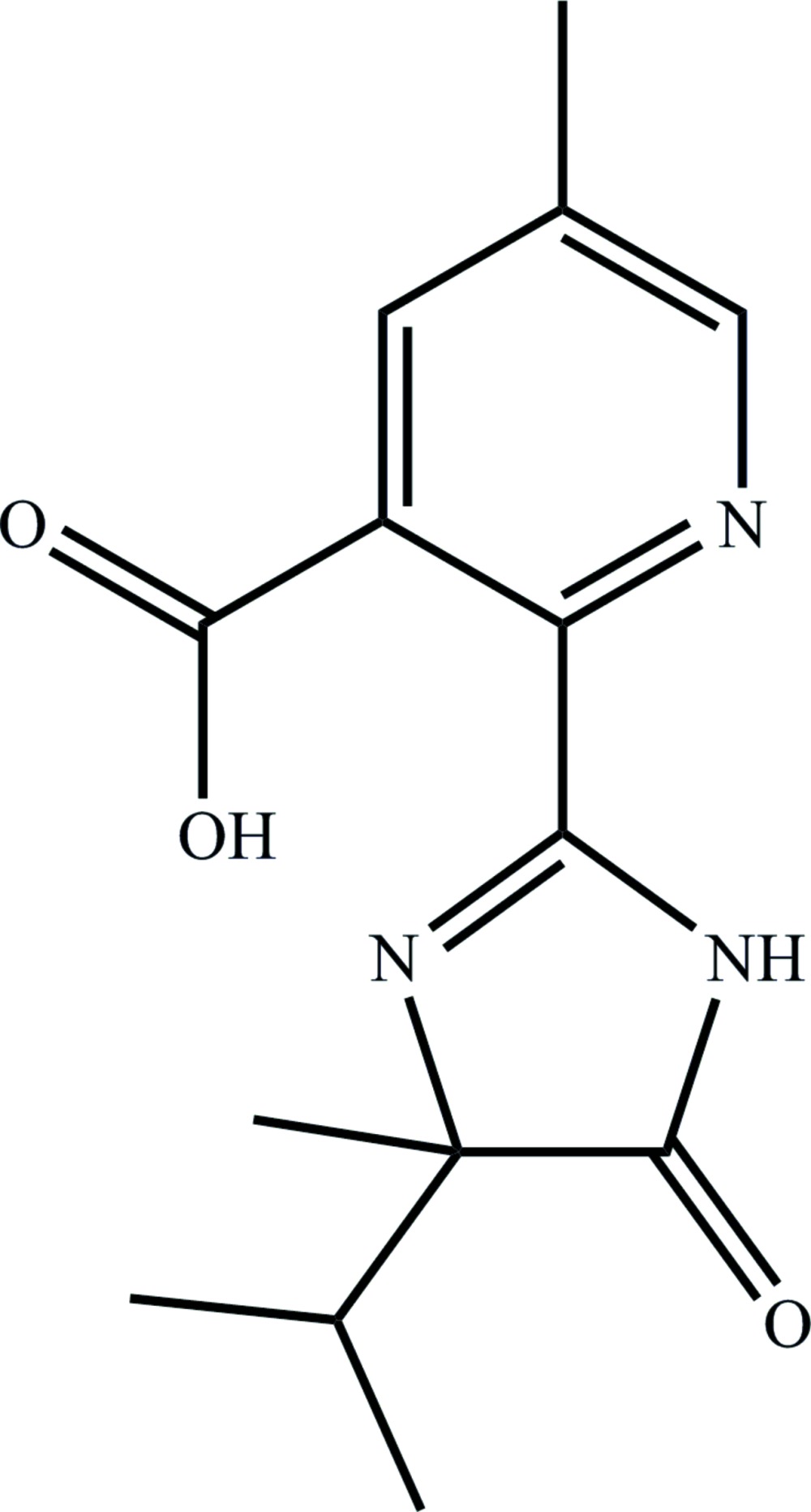


## Experimental

### 

#### Crystal data

C_14_H_17_N_3_O_3_
*M* *_r_* = 275.31
Monoclinic, 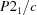

*a* = 12.102 (2) Å
*b* = 16.035 (3) Å
*c* = 7.2883 (15) Å
β = 94.17 (3)°
*V* = 1410.6 (5) Å^3^
*Z* = 4
Mo *K*α radiation
μ = 0.09 mm^−1^
*T* = 293 K
0.60 × 0.30 × 0.18 mm

#### Data collection

Rigaku R-AXIS RAPID diffractometer
Absorption correction: multi-scan (*ABSCOR*; Higashi, 1995 ▶) *T*_min_ = 0.946, *T*_max_ = 0.984
13471 measured reflections
3202 independent reflections
2234 reflections with *I* > 2σ(*I*)
*R*_int_ = 0.033

#### Refinement

*R*[*F* ^2^ > 2σ(*F* ^2^)] = 0.046
*wR*(*F* ^2^) = 0.145
*S* = 1.00
3202 reflections
192 parameters
2 restraints
H atoms treated by a mixture of independent and constrained refinement
Δρ_max_ = 0.28 e Å^−3^
Δρ_min_ = −0.17 e Å^−3^

### 

Data collection: *RAPID-AUTO* (Rigaku, 1998 ▶); cell refinement: *RAPID-AUTO*; data reduction: *CrystalClear* (Rigaku/MSC, 2002 ▶); program(s) used to solve structure: *SHELXS97* (Sheldrick, 2008 ▶); program(s) used to refine structure: *SHELXL97* (Sheldrick, 2008 ▶); molecular graphics: *SHELXTL* (Sheldrick, 2008 ▶); software used to prepare material for publication: *SHELXL97*.

## Supplementary Material

Crystal structure: contains datablock(s) I, global. DOI: 10.1107/S1600536812020478/ng5267sup1.cif

Structure factors: contains datablock(s) I. DOI: 10.1107/S1600536812020478/ng5267Isup2.hkl

Supplementary material file. DOI: 10.1107/S1600536812020478/ng5267Isup3.cml

Additional supplementary materials:  crystallographic information; 3D view; checkCIF report

## Figures and Tables

**Table 1:** Hydrogen-bond geometry (Å, °)

*D*—H⋯*A*	*D*—H	H⋯*A*	*D*⋯*A*	*D*—H⋯*A*
O1—H1⋯N2	0.82 (1)	1.68 (1)	2.4972 (16)	173 (2)
N3—H3⋯O2^i^	0.90 (1)	2.06 (1)	2.9387 (18)	165 (2)

Symmetry code: (i) 
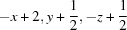
.

**Fig. 1.**
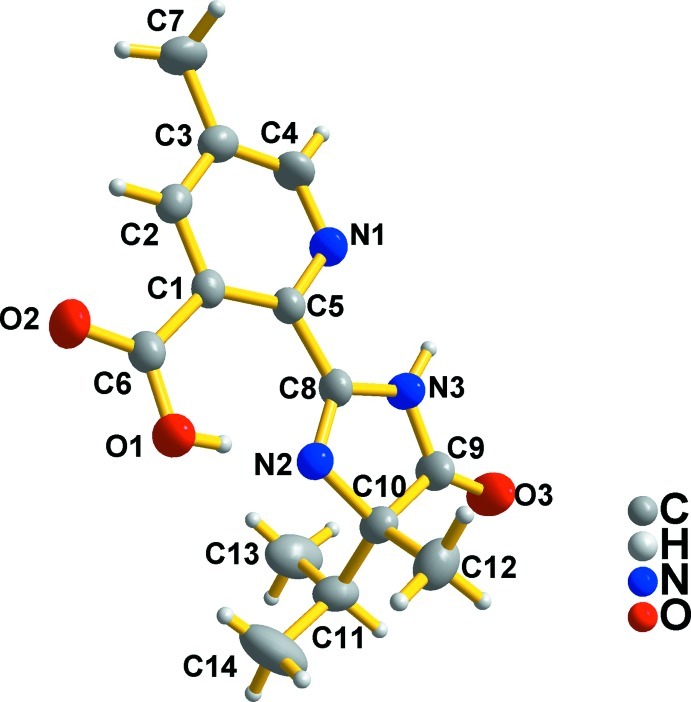
The molecular structure of the title compound, showing displacement ellipsoids at the 50% probability level for non-H atoms.

**Fig. 2.**
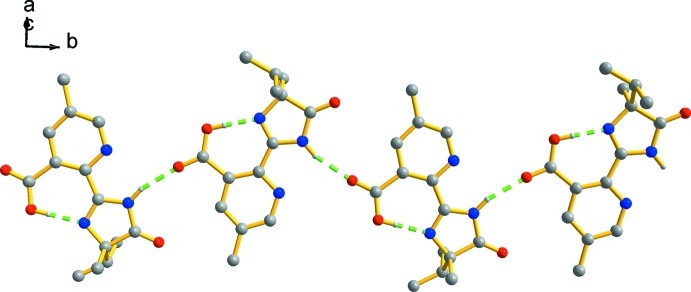
A partial packing view, showing the hydrogen-bonding chain structure along [010].

## supplementary crystallographic information

### Comment

Imazapic is an effective and widely used herbicide. Imazapic for the control of annual broadleaf and gramineae weeds has important achievements in agriculture. Herein, we report the crystal structure of this herbicide (Scheme I).

The pyridine and imidazole ring are almost coplanar with small dihedral angle of 3.08 (5) ° (Figure 1). There is an intramolecular O—H···N hydrogen bond; an intermolecular N—H···O hydrogen bond links isolated molecules into chain structure along [010] (Figure 2, Table 1).

### Experimental

The title compound was prepared by the reaction of diethyl 5-methylpyridine-2,3-dicarboxylate and 2-amino-2,3-dimethylbutanehydrazide according to a method reported in the patent literature. A white powder was obtained in 78% yield (Szezepanski *et al.*, 1988). Colorless crystals were obtained by the recrystallization of title compound from acetonitrile.

### Refinement

H atoms bound to C atoms were placed in calculated positions and treated as riding on their parent atoms, with C—H = 0.93 / 0.98 / 0.96 Å (aromatic / methine / methyl), and with *U*_iso_(H) = 1.2 / 1.5 *U*_eq_(C). N-bound and O-bound H atoms were located in a differece Fourier map and was refined with restraint as N—H = 0.90±0.01 Å and O—H = 0.82±0.01 Å, respectively, and *U*_iso_(H) = 1.5 *U*_eq_(O).

### Crystal data

**Table:**

C_14_H_17_N_3_O_3_	*F*(000) = 584
*M**_r_* = 275.31	*D*_x_ = 1.296 Mg m^−^^3^
Monoclinic, *P*2_1_/*c*	Mo *K*α radiation, λ = 0.71073 Å
Hall symbol: -P 2ybc	Cell parameters from 10539 reflections
*a* = 12.102 (2) Å	θ = 3.1–27.4°
*b* = 16.035 (3) Å	µ = 0.09 mm^−^^1^
*c* = 7.2883 (15) Å	*T* = 293 K
β = 94.17 (3)°	Block, colorless
*V* = 1410.6 (5) Å^3^	0.60 × 0.30 × 0.18 mm
*Z* = 4	

### Data collection

**Table:**

Rigaku R-AXIS RAPID diffractometer	3202 independent reflections
Radiation source: fine-focus sealed tube	2234 reflections with *I* > 2σ(*I*)
Graphite monochromator	*R*_int_ = 0.033
ω scan	θ_max_ = 27.5°, θ_min_ = 3.1°
Absorption correction: multi-scan (*ABSCOR*; Higashi, 1995)	*h* = −15→15
*T*_min_ = 0.946, *T*_max_ = 0.984	*k* = −20→20
13471 measured reflections	*l* = −9→9

### Refinement

**Table:**

Refinement on *F*^2^	Primary atom site location: structure-invariant direct methods
Least-squares matrix: full	Secondary atom site location: difference Fourier map
*R*[*F*^2^ > 2σ(*F*^2^)] = 0.046	Hydrogen site location: inferred from neighbouring sites
*wR*(*F*^2^) = 0.145	H atoms treated by a mixture of independent and constrained refinement
*S* = 1.00	*w* = 1/[σ^2^(*F*_o_^2^) + (0.0979*P*)^2^] where *P* = (*F*_o_^2^ + 2*F*_c_^2^)/3
3202 reflections	(Δ/σ)_max_ = 0.001
192 parameters	Δρ_max_ = 0.28 e Å^−^^3^
2 restraints	Δρ_min_ = −0.17 e Å^−^^3^

### Special details

**Table:**

Geometry. All esds (except the esd in the dihedral angle between two l.s. planes) are estimated using the full covariance matrix. The cell esds are taken into account individually in the estimation of esds in distances, angles and torsion angles; correlations between esds in cell parameters are only used when they are defined by crystal symmetry. An approximate (isotropic) treatment of cell esds is used for estimating esds involving l.s. planes.
Refinement. Refinement of F^2^ against ALL reflections. The weighted R-factor wR and goodness of fit S are based on F^2^, conventional R-factors R are based on F, with F set to zero for negative F^2^. The threshold expression of F^2^ > 2sigma(F^2^) is used only for calculating R-factors(gt) etc. and is not relevant to the choice of reflections for refinement. R-factors based on F^2^ are statistically about twice as large as those based on F, and R- factors based on ALL data will be even larger.

### Fractional atomic coordinates and isotropic or equivalent isotropic displacement parameters (Å^2^)

**Table:**

	*x*	*y*	*z*	*U*_iso_*/*U*_eq_	
C1	1.01695 (11)	0.78025 (8)	0.26937 (16)	0.0326 (3)	
C2	1.12083 (12)	0.76017 (9)	0.35266 (18)	0.0377 (3)	
H2	1.1400	0.7043	0.3680	0.045*	
C3	1.19647 (12)	0.82072 (10)	0.41336 (19)	0.0414 (3)	
C4	1.16383 (14)	0.90226 (10)	0.3862 (2)	0.0502 (4)	
H4	1.2136	0.9439	0.4251	0.060*	
C5	0.99292 (11)	0.86611 (8)	0.25086 (18)	0.0343 (3)	
C6	0.94567 (12)	0.70446 (8)	0.2115 (2)	0.0380 (3)	
C7	1.30826 (14)	0.79919 (12)	0.5050 (2)	0.0574 (5)	
H7A	1.3650	0.8150	0.4264	0.086*	
H7B	1.3122	0.7402	0.5275	0.086*	
H7C	1.3189	0.8286	0.6197	0.086*	
C8	0.88794 (12)	0.90408 (8)	0.17407 (18)	0.0364 (3)	
C9	0.77171 (13)	1.00954 (9)	0.1004 (2)	0.0463 (4)	
C10	0.71625 (13)	0.92605 (9)	0.0469 (2)	0.0403 (3)	
C11	0.60962 (13)	0.91307 (11)	0.1470 (2)	0.0502 (4)	
H11	0.5570	0.9565	0.1037	0.060*	
C12	0.69487 (16)	0.92380 (12)	−0.1627 (2)	0.0565 (5)	
H12A	0.7637	0.9304	−0.2187	0.085*	
H12B	0.6455	0.9683	−0.2015	0.085*	
H12C	0.6620	0.8713	−0.1991	0.085*	
C13	0.62931 (18)	0.92271 (16)	0.3541 (2)	0.0755 (6)	
H13A	0.6825	0.8821	0.4004	0.113*	
H13B	0.5609	0.9145	0.4104	0.113*	
H13C	0.6570	0.9777	0.3823	0.113*	
C14	0.5557 (2)	0.82921 (17)	0.1010 (4)	0.0935 (8)	
H14A	0.6040	0.7852	0.1465	0.140*	
H14B	0.5424	0.8240	−0.0299	0.140*	
H14C	0.4866	0.8255	0.1576	0.140*	
N1	1.06558 (11)	0.92563 (7)	0.30766 (19)	0.0475 (3)	
N2	0.80181 (10)	0.86483 (7)	0.10681 (16)	0.0382 (3)	
N3	0.87614 (11)	0.98947 (8)	0.17332 (19)	0.0459 (3)	
H3	0.9282 (14)	1.0274 (10)	0.208 (3)	0.066 (5)*	
O1	0.85037 (10)	0.71349 (6)	0.12055 (16)	0.0499 (3)	
H1	0.8310 (18)	0.7624 (4)	0.109 (3)	0.075*	
O2	0.98114 (10)	0.63550 (7)	0.25044 (19)	0.0606 (4)	
O3	0.73227 (11)	1.07853 (7)	0.0816 (2)	0.0687 (4)	

### Atomic displacement parameters (Å^2^)

**Table:**

	*U*^11^	*U*^22^	*U*^33^	*U*^12^	*U*^13^	*U*^23^
C1	0.0366 (7)	0.0277 (6)	0.0341 (6)	0.0013 (5)	0.0064 (5)	0.0019 (5)
C2	0.0377 (8)	0.0335 (7)	0.0422 (7)	0.0061 (6)	0.0051 (6)	0.0037 (5)
C3	0.0362 (8)	0.0468 (8)	0.0412 (7)	0.0022 (6)	0.0021 (6)	0.0027 (6)
C4	0.0417 (9)	0.0429 (9)	0.0641 (9)	−0.0103 (7)	−0.0101 (7)	0.0015 (7)
C5	0.0353 (7)	0.0283 (6)	0.0392 (7)	−0.0019 (5)	0.0021 (5)	0.0023 (5)
C6	0.0399 (8)	0.0271 (7)	0.0474 (7)	0.0013 (6)	0.0054 (6)	−0.0007 (5)
C7	0.0376 (9)	0.0683 (12)	0.0649 (10)	0.0034 (8)	−0.0059 (7)	0.0029 (8)
C8	0.0389 (8)	0.0257 (7)	0.0444 (7)	−0.0004 (5)	0.0019 (6)	0.0017 (5)
C9	0.0439 (9)	0.0318 (8)	0.0625 (9)	0.0029 (6)	−0.0005 (7)	0.0021 (6)
C10	0.0389 (8)	0.0338 (7)	0.0470 (8)	0.0030 (6)	−0.0049 (6)	0.0013 (6)
C11	0.0362 (8)	0.0590 (10)	0.0543 (9)	−0.0013 (7)	−0.0032 (6)	−0.0023 (7)
C12	0.0643 (11)	0.0590 (11)	0.0454 (8)	0.0105 (8)	−0.0020 (7)	0.0053 (7)
C13	0.0623 (13)	0.1113 (19)	0.0536 (10)	−0.0148 (11)	0.0090 (9)	−0.0095 (10)
C14	0.0753 (16)	0.1046 (18)	0.1019 (17)	−0.0496 (14)	0.0153 (13)	−0.0230 (14)
N1	0.0431 (8)	0.0325 (7)	0.0652 (8)	−0.0056 (5)	−0.0082 (6)	0.0031 (6)
N2	0.0370 (7)	0.0290 (6)	0.0478 (6)	−0.0004 (5)	−0.0033 (5)	0.0015 (5)
N3	0.0406 (7)	0.0266 (6)	0.0689 (8)	−0.0005 (5)	−0.0075 (6)	−0.0002 (5)
O1	0.0463 (6)	0.0279 (5)	0.0737 (7)	−0.0019 (5)	−0.0094 (5)	−0.0031 (5)
O2	0.0548 (7)	0.0265 (5)	0.0989 (9)	0.0039 (5)	−0.0055 (6)	0.0038 (5)
O3	0.0566 (8)	0.0315 (6)	0.1157 (11)	0.0103 (5)	−0.0100 (7)	0.0003 (6)

### Geometric parameters (Å, º)

**Table:**

C1—C2	1.393 (2)	C9—N3	1.373 (2)
C1—C5	1.4116 (18)	C9—C10	1.535 (2)
C1—C6	1.5317 (19)	C10—N2	1.4699 (17)
C2—C3	1.385 (2)	C10—C12	1.531 (2)
C2—H2	0.9300	C10—C11	1.542 (2)
C3—C4	1.376 (2)	C11—C13	1.519 (2)
C3—C7	1.505 (2)	C11—C14	1.521 (3)
C4—N1	1.335 (2)	C11—H11	0.9800
C4—H4	0.9300	C12—H12A	0.9600
C5—N1	1.3426 (18)	C12—H12B	0.9600
C5—C8	1.4813 (19)	C12—H12C	0.9600
C6—O2	1.2124 (17)	C13—H13A	0.9600
C6—O1	1.2959 (19)	C13—H13B	0.9600
C7—H7A	0.9600	C13—H13C	0.9600
C7—H7B	0.9600	C14—H14A	0.9600
C7—H7C	0.9600	C14—H14B	0.9600
C8—N2	1.2840 (18)	C14—H14C	0.9600
C8—N3	1.3767 (18)	N3—H3	0.898 (9)
C9—O3	1.2087 (18)	O1—H1	0.8203 (10)
			
C2—C1—C5	116.11 (12)	N2—C10—C11	111.41 (12)
C2—C1—C6	114.13 (12)	C12—C10—C11	112.47 (13)
C5—C1—C6	129.76 (12)	C9—C10—C11	111.28 (13)
C3—C2—C1	122.12 (13)	C13—C11—C14	110.02 (17)
C3—C2—H2	118.9	C13—C11—C10	112.34 (14)
C1—C2—H2	118.9	C14—C11—C10	112.06 (15)
C4—C3—C2	116.36 (13)	C13—C11—H11	107.4
C4—C3—C7	121.43 (15)	C14—C11—H11	107.4
C2—C3—C7	122.21 (15)	C10—C11—H11	107.4
N1—C4—C3	124.46 (14)	C10—C12—H12A	109.5
N1—C4—H4	117.8	C10—C12—H12B	109.5
C3—C4—H4	117.8	H12A—C12—H12B	109.5
N1—C5—C1	122.55 (13)	C10—C12—H12C	109.5
N1—C5—C8	110.42 (12)	H12A—C12—H12C	109.5
C1—C5—C8	127.01 (12)	H12B—C12—H12C	109.5
O2—C6—O1	120.56 (13)	C11—C13—H13A	109.5
O2—C6—C1	118.47 (13)	C11—C13—H13B	109.5
O1—C6—C1	120.97 (11)	H13A—C13—H13B	109.5
C3—C7—H7A	109.5	C11—C13—H13C	109.5
C3—C7—H7B	109.5	H13A—C13—H13C	109.5
H7A—C7—H7B	109.5	H13B—C13—H13C	109.5
C3—C7—H7C	109.5	C11—C14—H14A	109.5
H7A—C7—H7C	109.5	C11—C14—H14B	109.5
H7B—C7—H7C	109.5	H14A—C14—H14B	109.5
N2—C8—N3	113.88 (12)	C11—C14—H14C	109.5
N2—C8—C5	126.36 (12)	H14A—C14—H14C	109.5
N3—C8—C5	119.75 (12)	H14B—C14—H14C	109.5
O3—C9—N3	127.10 (15)	C4—N1—C5	118.40 (13)
O3—C9—C10	127.42 (15)	C8—N2—C10	108.71 (11)
N3—C9—C10	105.48 (12)	C9—N3—C8	109.09 (12)
N2—C10—C12	110.19 (13)	C9—N3—H3	123.8 (13)
N2—C10—C9	102.79 (11)	C8—N3—H3	127.1 (14)
C12—C10—C9	108.25 (13)	C6—O1—H1	113.3 (16)

### Hydrogen-bond geometry (Å, º)

**Table:**

*D*—H···*A*	*D*—H	H···*A*	*D*···*A*	*D*—H···*A*
O1—H1···N2	0.82 (1)	1.68 (1)	2.4972 (16)	173 (2)
N3—H3···O2^i^	0.90 (1)	2.06 (1)	2.9387 (18)	165 (2)

Symmetry code: (i) −*x*+2, *y*+1/2, −*z*+1/2.
